# Investigation of *Clostridium perfringens* in small-scale commercial broiler flocks in Mymensingh district of Bangladesh

**DOI:** 10.14202/vetworld.2021.2809-2816

**Published:** 2021-10-29

**Authors:** Arunima Oarin Tresha, Mohammad Arif, Sk Shaheenur Islam, A. K. M. Ziaul Haque, Md. Tanvir Rahman, S. M. Lutful Kabir

**Affiliations:** Department of Microbiology and Hygiene, Bangladesh Agricultural University, Mymensingh, Bangladesh.

**Keywords:** antimicrobial-resistant, Bangladesh, broiler, *Clostridium perfringens*, necrotic enteritis, prevalence

## Abstract

**Background and Aim:**

Necrotic enteritis (NE) is one of the most prevalent diseases in broiler poultry caused by *Clostridium perfringens* connected with significant economic losses. A cross-sectional survey was conducted in Mymensingh district of Bangladesh to assess the prevalence of *C. perfringens* through toxinotyping molecular assay and confirm the risk factors for NE, including antimicrobial-resistant (AMR) status of the isolates.

**Materials and Methods:**

We included 40 small-scale commercial broiler farms randomly selected from two subdistricts of Mymensingh district of Bangladesh. As an individual sample, 240 cloacal swabs, and as a pooled sample, 40 drinking water, 40 workers’ hand washing, 40 litter swab, and 40 feed samples were collected and evaluated by culture, biochemical, and molecular assays. A pretested semi-structured interview questionnaire was employed to capture flock-level data on risk factors from the farm owners. The flock-level data on risk factors were assessed through univariable and multivariable logistic regression analyses with p<0.05 was considered statistically significant.

**Results:**

Overall flock-level prevalence of *C. perfringens* was estimated to be 10.3% (95% confidence interval [CI] 7.5-13.6%). Litter swab (pooled) was found to be highly contaminated with *C. perfringens* (25.0%, 95% CI: 12.7-41.2%) followed by the cloacal swab (10.4%, 95% CI: 6.9-15.0%) and feed sample (5.0%, 95% CI: 0.6-16.9%). History of coccidia infection (Adjusted odds ratio =33.01, 95% CI: 2.14-507.59, p=0.01) was significantly associated with flock-level *C. perfringen*s infection status. In this study, 78.1% isolates were found as multidrug-resistant as they demonstrated resistance to 3-5 antimicrobial agents.

**Conclusion:**

Evidence-based control options need to be taken through the uses of prebiotics and probiotics, biosecurity, and hygienic measurement, including control of coccidia infection, is needed to lessen the NE infection and AMR related to this pathogen in small-scale commercial broiler poultry.

## Introduction

*Clostridium perfringens* is a Gram-positive, anaerobic, spore-forming pathogen [1]. This organism is usually present in the gastrointestinal tract of animals and humans. However, its ubiquitous distribution in the environments, especially in soil, sewage, feces, and sediments which could facilitate horizontal transmission [1,2]. *C. perfringens* causes many diseases both in humans and animals, including gas gangrene and food poisoning in humans and necrotic enteritis (NE) in poultry species [3].

NE is considered to be one of the highly prevalent diseases of broiler chickens that induce a huge economic burden for poultry industry all over the world. This disease incurs an estimated economic loss between 2 and 6 billion USD per annum [4,5]. Due to the occurrence of this disease, body weight reduced by 12% and feed conversion ratio increased by 11% in comparison to the healthy flocks.

The disease can occur between 2 and 6 weeks of age in broiler birds, both in clinical and subclinical forms [6]. Most of the clinical form of NE in chicken is characterized by unexpected high mortality without any remarkable signs; however, weight loss and poor feed conversion are noticed in subclinical form [7]. Several determinants namely, non-starch polysaccharide-enriched feed (wheat and barley), presence of coccidiosis in the flock, and inclusion of fish meal protein in the poultry feed are responsible for the occurrence of *C. perfringen*s infection in poultry flock [8-11].

*C. perfringens* is divided into five classes, namely, A, B, C, D, and E; of which A, C, and D have been documented to cause NE in chickens [12,13]. The pathogenicity of *C. perfringens* is connected with the production of several toxins, for example, alpha (α), beta (β), epsilon (ε), and iota (ι) toxins [14,15]. The major infection in poultry is caused by type “A” and with a lesser extent by type “C” strains of *C. perfringens*. Type “A” strain produces α-toxin; however, type “C” produces α-toxin and β-toxin. The α-toxin produced by *C. perfringens* is considered to be the major virulence factor in the pathogenesis of NE [16,17]. In addition, two new toxinotypes have been identified, namely, *C. perfringens* type F and G. Among them, *C. perfringens* type F causes food poisoning and antibiotic-related gastroenteritis in humans. *C. perfringens* type G generates NetB toxin and thus causes NE in poultry [18] which have been established to play the key role in the pathogenesis of NE [19,20].

In Bangladesh, small-scale commercial poultry farms contribute 50-60% poultry meat to the national demand [21] and chicken from this production system is more likely to be colonization with infectious pathogens, even *C. perfringens* due to lower level of biosecurity measures. A few studies were conducted in Bangladesh to explore the burden of *C. perfringens* sparsely from NE suspected chickens [22-24]. However, none of the studies employed molecular-based robust technique (polymerase chain reaction [PCR]) and to evaluate antimicrobial susceptibility status along with confirmation of risk factors.

Therefore, this study was carried out to investigate the prevalence, risk factors, molecular detection, and antimicrobial resistance status of C. *perfringens* in small-scale commercial broiler farms in Mymensingh district of Bangladesh. The outcomes of this epidemiological survey highlight the control options for broiler diseases connected with this pathogen together with providing the data for public health and food safety risk assessment.

## Materials and Methods

### Ethical approval and Informed consent

The study was approved by the Animal Welfare and Experimentation Ethics Committee (AWEEC) of Bangladesh Agricultural University under reference no. AWEEC/BAU/2021(12). The broiler farms were chosen after discussion with the subdistrict (Upazila) livestock offices of Department of Livestock Services, Bangladesh. Verbal consent was obtained from each of the farmers during field interview data collection and subsequent poultry sampling as a substantial number of poultry farmers are illiterate and cannot read and write.

### Study period and location

**Figure-1:**
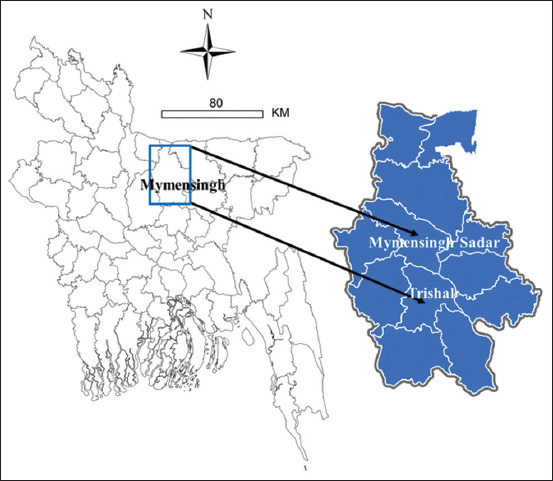
Locations of the surveyed farms in two subdistricts (Mymensingh Sadar and Trishal) of Mymensingh district of Bangladesh indicated by arrows on the map. The map was produced in ArcGIS-ArcMap version 10.3 (ESRI Co., Redlands, California, USA) using geographic coordinates of the study locations captured via Garmin eTrex 10. A total of 40 broiler farms were surveyed that represented equal number of farms were included from each subdistrict with a flock size >3000 birds under sector three production systems [Source: Map was prepared using ArcGIS-ArcMap software version 10.3].

The study was conducted from September 2019 to May 2020. The study was conducted in two subdistricts (Mymensingh Sadar and Trishal) of Mymensingh district under a cross-sectional survey (Figure-1).

### Study design and location of poultry farms

The study used 40 broiler farms that included an equal number of farms from each subdistrict. The farms were selected with an inclusion criterion of minimum flock size >3000 under small-scale commercial (sector three) poultry production system at a lower level of biosecurity standard with apparently healthy birds.

### Sample collection and shipment

An equal number of samples (both environmental and individual poultry samples) were randomly collected from each broiler farm. All samples collected from each farm were recorded in a sample collection checklist. A total of 10 samples were collected from each farm, of which, six samples were collected as environmental samples (drinking water [n=1], feed [n=1], workers handwashing [n=1], and litter swab [n=1]) and six cloacal swab samples (single cloacal swab from each bird) were collected from each apparently healthy flock. As an environmental sample, three samples were collected from each farm and pooled together as an environmental sample for the category. The amount of samples was varied according to the category of samples as 200 mL water, 100 g feed, 200 mL workers hand washing, and 1-5 mL or mg litter/cloacal swab materials. The swab samples were preserved in normal saline. All samples were retained in sterile zipper bags and labeled with a unique identification code. The samples were transported to the designated laboratory with maintaining a cool chain as early as possible after collection (4-6°C).

### Isolation and Identification

#### Culture and biochemical tests

The samples were processed immediately after arrival at the designated laboratory as per the standard method [25]. Isolation and identification of *C. perfringens* were accomplished as per the protocol described earlier [26]. In brief, initial enrichment was made using the processed sample in Robertson’s Cooked Meat media (RCM broth), (HiMedia, Mumbai, India), subsequently, anaerobic incubation at 37°C for 24 h through candle jar method. The existence of turbidity in the incubated broth media implies presence of anaerobic bacteria. A loopful of positive culture was streaked on perfringens agar base (TSC; HiMedia, India) with selective supplement (HiMedia) and incubated in the anaerobic jar at 37°C for 24 h. Successively, cultured plates were observed for the growth of *C. perfringens* and colony morphology was evaluated and recorded concurrently. Gram’s staining, motility test through hanging drop method, and biochemical tests such as catalase and oxidase tests were accomplished as per standard protocol [27]. Thus, obtained pure colonies were used in the molecular evaluation.

### Molecular detection

In this evaluation, DNA was extracted from the pure bacterial culture by boiling method as previously described [28-30]. In brief, a loopful of bacterial colonies was taken and suspended in 1.5 mL microcentrifuge tubes containing 200 μL distilled water by gentle vortexing and then centrifuged for 10 min at 14,000× *g*. After that, the pellet was suspended in 200 μL of TE buffer by gentle vortexing. Then, the microcentrifuge tubes were boiled for 15 min at 100°C and immediately chilled on ice for 10 min. An aliquot of 50 μL of the supernatant was stored as the DNA template for PCR. PCR amplification was carried out to detect toxin typing (alpha, beta, epsilon, and iota toxin genes of *C. perfringens* through multiplex reaction). Specific oligonucleotide primer sequences and corresponding to alpha, beta, epsilon, and iota toxin genes of *C. perfringens* were used in this study [14]. The list of primers with thermal conditions utilized in PCR is shown in Table-1.

**Table-1:** The list of primers with thermal conditions used in this study.

Primer	Sequence(5’-3’)	Target	Amplicon size	PCR condition (35 cycles)			Reference

				Denature	Annealing	Extension	
Cpa-F	GTTGATAGCGCAGGACATGTTAAG	*cpa* (Alpha toxin)	402 bp	94°C, 30 s	59°C, 45 s	72°C, 45 s	[14]
Cpa-R	CATGTAGTCATCTGTTCCAGCATC						
Cpb-F	ACTATACAGACAGATCATTCAACC	*cpb* (Beta toxin)	236 bp	94°C, 30 s	59°C, 45 s	72°C, 45 s	
Cpb-R	TTAGGAGCAGTTAGAACTACAGAC						
Etx-F	ACTGCAACTACTACTCATACTGTG	*Etx* (Epsilon toxin)	541 bp	94°C, 30 s	59°C, 45 s	72°C, 45 s	
Etx-R	CTGGTGCCTTAATAGAAAGACTCC						
Cpi-F	GCGATGAAAAGCCTACACCACTAC	*cpi* (Iota toxin)	317 bp	94°C, 30 s	59°C, 45 s	72°C, 45 s	
Cpi-R	GGTATATCCTCCACGCATATAGTC						

PCR=Polymerase chain reaction

The PCR assay was accomplished using a thermal cycler (Thermo Cycler, ASTEC, Japan) following the protocols described earlier [31]. Each multiplex PCR reaction mix (for alpha, beta, epsilon, and iota toxins) contained 8 μL of extracted DNA template from bacterial cultures, 25 μL PCR master mix (Promega, USA), 1 μL of each alpha, beta, epsilon, and iota forward and reverse primers (20 pmol/μL), and 9 μl of PCR-grade water, to form a total volume of 50 μL in a PCR tube for amplification.

The PCR products were pictured in gel electrophoresis (1.5-2% agarose, Invitrogen, Carlsbad, CA, USA), and further, colored with ethidium bromide (0.5 g/mL) and decolored with distilled water, 10 min for each step, before gel images were taken using an ultraviolet transilluminator (Biometra, Göttingen, Germany).

### Antimicrobial susceptibility testing

All strains of *C. perfringens* were evaluated against seven commercially available antimicrobials in Bangladesh, such as amoxicillin (AMX 30 μg), ciprofloxacin (CIP 5 μg), chloramphenicol (CHL 30 μg), erythromycin (ERY 15 μg), gentamicin (GEN 10 μg), oxytetracycline (OTE 30 μg), and ceftriaxone (CRO 30 μg) (HiMedia) through disk diffusion method. The zones of growth inhibition connected with the zone diameter were interpreted as per standards as labeled by the Clinical and Laboratory Standard Institute [32], and therefore, concluded as susceptible (S), intermediate resistant (I), or resistant (R) to the tested antimicrobial. *Escherichia coli* strain ATCC 25922 was used as a quality control organism. In this assessment, all interpretations were confirmed by the completion of at least two duplicates of the disk diffusion test.

### Data collection on risk factors

A pretested semi-structured questionnaire was developed in English containing 15 closed-ended questions. The questionnaire mainly focused on farm management and biosecurity-related parameters. The questionnaire was translated into local language so that the farmers can easily understand its content. Two expert veterinarians and one trained enumerator were involved at face-to-face interview data collection process.

### Data management and statistical analyses

The data for laboratory test and interview data were taken in hard copies and further recorded in Microsoft Excel^®^ spreadsheets. Data were imported into Epi Info 7 program (Centers for Disease Control and Prevention, Georgia, USA) [33] for statistical analysis. The categorical data were shown as frequencies and proportion, and 95% binomial confidence intervals (CIs) were confirmed using the Excel data analysis tool pack. The odds ratio (OR) was calculated through a univariate logistic regression model for estimating the relationship on flock level *C. perfringens* positive status, and p<0.05 was was considered statistically significant, finally, significant variables were included in the multivariable logistic regression analysis.

## Results

### Prevalence of *C. perfringens*

The survey evaluated an overall prevalence of C*. perfringens* as 10.3% (95% CI: 7.5-13.6), including all categories of samples through culture, biochemical tests, and finally, toxinotyping molecular detection. Among the environment samples, litter swab was found to be highly contaminated with C*. perfringens* (25.0%, 95% CI: 12.7-41.2) followed by worker’s handwashing and poultry feed, respectively. However, a prevalence of 10.4% (95% CI: 6.9-15.0) was estimated in poultry swab samples. The distribution of prevalence in different samples was found to be statistically significant (p=0.004) (Table-2). Of 40 farms, 24 were found contaminated with C*. perfringens*; therefore, 60% (95% CI: 43.3%-75.1) prevalence was observed at the farm level.

**Table-2:** Prevalence of *C. perfringens* in broiler farms of Mymensingh district of Bangladesh.

Sample category	Number of samples tested	Number of positive sample	Prevalence (95% CI)	p-value
Environmental samples				0.004
Drinking water	40	0	0.0 (0.0-8.8)	
Worker’s hand washing	40	4	10.0 (2.8-23.7)	
Litter swab	40	10	25.0(12.7-41.2)	
Feed	40	2	5.0(0.6-17.0)	
Poultry samples				
Cloacal swab	240	25	10.4 (6.9-15.0)	
Overall	400	41	10.3 (7.5-13.6)	

*C. perfringens=Clostridium perfringens*

### Molecular detection of the isolates

**Figure-2:**
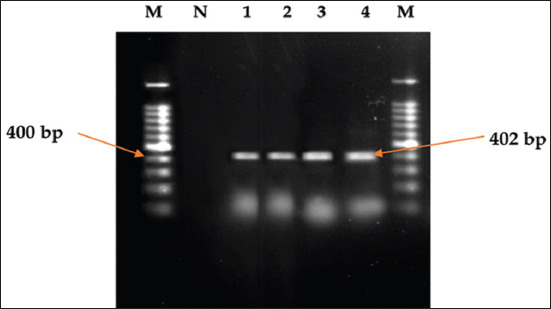
Agarose gel electrophoresis showing 402 bp amplicon of alpha-toxin gene (*cpa* gene) of *Clostridium perfringens* [14]. Lane M: 100 bp DNA marker; lane N: Negative control; lanes 1-4: Isolates positive for *C. perfringens*.

All 41 isolates were utilized in toxinotyping *cpa, cpb*, *etx*, and *cpi* gene-based multiplex PCR and found positive only for *cpa* as they demonstrated 402 bp amplicon of alpha-toxin gene (*cpa* gene) of *C. perfringens* (Figure-2). Thus, isolates were confirmed as toxinotype A. None of the isolates carried *cpb, etx*, or *cpi* genes and demonstrated the absence of *C. perfringens* toxinotype B, C, D, or E in positive samples.

### Risk factors assessment

A total of 15 parameters regarding farm management, biosecurity practices were used in univariable analysis. In this analysis, three variables such as age of the birds (weeks), history of coccidia infection, and litter type were significantly related with the flock-level *C. perfringens* positive status. The rest of the factors were found to be non-significant with the flock-level outcome status (Table-3).

**Table-3:** Result from univariable logistic regression analysis displaying the relationship with farm level *C. perfringens* infection in 40 broiler farms.

Variables	Category	Positive (%)	Odds ratio	p-value
Age of the birds (weeks)	1-3 weeks (n=24)	18 (75.0)	Reference	0.04
	3-4 weeks (n=16)	6 (37.5)	5.0 (1.3-19.7)	
History of coccidia infection	Yes (n=19)	18 (94.7)	45 (4.9-416.5)	0.000
	No (n=21)	6 (28.6)	Reference	
Disposal of dead birds	Throw elsewhere (n=31)	18 (58.1)	1.4 (0.3-6.8)	0.64
	Burial (n=9)	6 (66.7)	Reference	
Feeder and drinker wash daily	Yes (n=14)	8 (57.1)	Reference	0.78
	No (n=26)	16 (66.7)	1.2 (0.3-4.5)	
Clean interval of litter	1/week (n=30)	16 (66.7)	0.8 (0.5-1.1)	0.13
	2/week (n=10)	8 (80.0)	Reference	
Litter type	Wet (n=15)	13 (86.7)	8.3 (1.5-44.6)	0.007
	Dry (n=25)	11 (44.0)	Reference	
Stocking density	1 Sq. feet/bird (n=9)	4 (44.4)	0.4 (0.09-2.0)	0.30
	> 1 Sq. feet/bird (n=31)	20 (83.3)	Reference	
Waste disposal of poultry farm	Within 10 m of far(n=38)	23 (60.5)	1.5 (0.1-26.4)	0.76
	> 10 m of the farm (n=2)	1 (50.0)	Reference	

*C. perfringens=Clostridium perfringens*

Among the risk factors, three were included in the multivariable mixed-effect logistic regression analysis as these were captured to be statistically significant in univariable analysis. The potential risk factors for flock-level *C. perfringens* were identified in this final model. The most important risk factor connected with the flock-level *C. perfringens* infection was identified as “history of coccidia infection in the flock” (Table-4). The likelihood of *C. perfringens* infection was found 33.01 times (95% CI: 2.14-507.59, p=0.01) higher in broiler farms with the history of coccidia infection.

**Table-4:** Factors retained in the final multivariable mixed-effect logistic regression model of risk of flock level *C. perfringens* infection.

Determinates	Category	AOR	95% CI	Coefficient	SE	Z-statistic	p-value
Age of the bird (weeks)	1-3	1.61	0.27-9.69	0.47	0.91	0.521	0.60
	3-4						
History of coccidia infection in the flock	Yes	33.01	2.14-507.59	3.49	1.39	2.5083	0.01
	No						
Litter condition	Wet	1.21	0.09-15.17	0.19	1.287	0.1525	0.87
	Dry						

AOR=Adjusted odds ratio, CI: Confidence interval, SE= Standard error. *C. perfringens=Clostridium perfringens*

### Antibiogram

#### Antimicrobial susceptibility pattern

In this assessment, of 41 isolates of *C. perfringens*, 92.7% (n=38) were documented S to CIP, followed by CRO (85.4%, n=35AMX (70.7%, n=29), CHL (36.6%, n=15), respectively. However, 29.3% (n=12) isolates were revealed intermediate S to OTE (29.3%, n=12), followed by CHL (22.0%, n=9), AMX (14.6%, n=6), and CIP and/or ERY (7.3%, n=3), respectively. Alarmingly, no isolates (0%, n=0) was captured as S or I-S to GEN (Table-5).

**Table-5:** Antimicrobial susceptibility status of *C. perfringens* isolates from 40 broiler flocks.

Antimicrobial agents	Isolates		

	Resistant % (n)	Intermediate % (n)	Susceptible % (n)
Amoxicillin (30 µg)	14.6 (6)	14.6 (6)	70.7 (29)
Chloramphenicol (30 µg)	41.5 (17)	22.0 (9)	36.6 (15)
Ciprofloxacin (5 µg)	0.0 (0)	7.3 (3)	92.7 (38)
Erythromycin (15 µg)	92.7 (38)	7.3 (3)	0.0 (0)
Gentamicin (10 µg)	100.0 (41)	0.0 (0)	0.0 (0)
Oxytetracycline (30 µg)	70.7 (29)	29.3 (12)	0.0 (0)
Ceftriaxone (30 µg)	0.0 (0)	14.6 (6)	85.4 (35)

*C. perfringens=Clostridium perfringens*

### Antimicrobial-resistance (AMR) status

**Figure-3:**
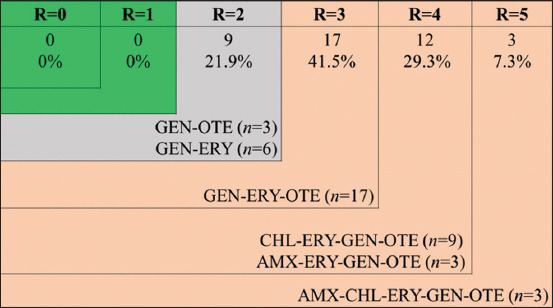
Distribution of antimicrobial resistance status for *Clostridium perfringens* (n=41) strains isolated from broiler farms, R=Number of antibiotic resistances; AMX=Amoxicillin, CHL=Chloramphenicol, ERY=Erythromycin, OTE=Oxytetracycline, GEN=Gentamicin, n=Number of isolates with the indicated pattern.

Among the 41 isolates of *C. perfringens*, 7.3% (n=3) and 14.6% (n=6) were demonstrated resistance to two antimicrobial agents, GEN-OTE and GEN-ERY, respectively. However, 41.5% (n=17) were shown R to three antimicrobial agents (GEN-ERY-OTE). Alarmingly, 22.0% (n=9) and 7.3% (n=3) of isolates were captured R to four antimicrobial agents, CHL-ERY-GEN-OTE and AMX-ERY-GEN-OTE, respectively, and 7.3% (n=3) to five antimicrobial agents (AMX-CHL-ERY-GEN-OTE) (Figure-3).

## Discussion

We evaluated prevalence, risk factors, molecular confirmation though toxinotyping gene and AMR pattern of *C. perfringens* isolated from small-scale commercial broiler flocks of a promising poultry rearing district of Bangladesh. In this survey, we evaluated *C. perfringens* in apparently healthy flocks through a-toxin gene-targeted primers. Thus, 41 isolates were established for *cpa* gene (402 bp) that signifies *C. perfringens* type A in the broiler samples. These PCR assays were also reported to be utilized by other researchers in India [28,31].

*C. perfringens* was found to be prevalent in 60% farms (24/40) with a sample level prevalence was documented as 10.3% (41/400) in apparently healthy flocks. These results can be elucidated by the view as a commensal organism, *C. perfringens* is living in the gastrointestinal tract of humans and animals [7,34]. In general, healthy birds have *C. perfringens* below 10^5^ CFU/gram intestinal content. Nevertheless, the increase in the number of *C. perfringens* between 10^7^ and 10^9^ CFU/gram intestinal content has been confirmed NE outbreaks in healthy poultry flocks [35].

This finding of this study is consistent with the similar study conducted in Tamil Nadu, India, as 10.76% prevalence of *C. perfringens* was confirmed in livestock and poultry specimens [36]. Other studies conducted in India captured higher prevalence as 33.89% in poultry feed samples in Tamil Nadu, South India [37], 33.63% and 18.91% in broiler of 2-6 weeks age and older layer, respectively, in Kashmir, India [38], and 53% dead broilers in West Bengal, India [39]. The prevalence that was established from this study is comparatively lower than the earlier studies conducted in different geographical locations 48.82% [40] and 23.1% [41] in China; 29.6% in Taiwan [42]; 57.9% in Egypt [29]; and 24.72% and 23.28% from two commercial poultry farms in Canada [43]. However, we could not compare our study findings in a country context due to the lack of reference data of NE in broiler chicken using the advance molecular assay. There are several studies conducted in Bangladesh that have confirmed the prevalence of *C. perfringens* between 0.4% to 1% based on history, clinical findings, including postmortem lesions [22-24].

The crude relationship between flock-level risk factors and *C. perfringens*-positive status was age of the birds (3-4 weeks), history of coccidia infection, and litter type (wet). The age of birds (3-4 weeks) (OR=5.0, 95% CI: 1.3-19.7, p=0.04) and wet litter (OR=8.3, 95% CI: 1.5-44.6, p=0.007) were found to be more likely with *C. perfringens* infection in broiler flocks. Similarly, history of coccidian infection (OR=45, 95% CI: 4.9-416.5, p=0.000) and wet litter (OR=8.3, 95 CI: 1.5-44.6, p=0.007) have more likelihood with the farm level *C. perfringens* infection. The association of the occurrence of *C. perfringens* with the age group (3-4 weeks) has been confirmed by another study in Egypt [29]. Similarly, litter conditions can play an important role for NE occurrence in the poultry flock, which is supported by another study [44]. However, in the final multivariable logistic model, history of coccidia infection (adjusted OR=33.01, 95% CI: 2.14-507.59, p=0.01) was found to be a strong association with *C. perfringens* infection in the broiler flocks. This finding is supported by other authors as the leading risk factor for NE, coccidia infection causes damage of the mucous membrane that supports to the attachment of *C. perfringens* [8,10]. Therefore, prevention of coccidial infection is needed to control NE outbreaks in the poultry flocks.

The antimicrobial sensitivity status in this study showed that *C. perfringens* isolates were 92.7% sensitive to CIP followed by CRO (85.4%) and AMX (70.7%). However, a lower level of susceptibility was detected to CHL (36.0%). These findings are partially corroborated by other studies [26,45-47]. Alarmingly, a few antimicrobials such as ERY, gentamycin, and OTE were captured as S to 0.0% of isolates. This finding indicates that some antimicrobials agents such as ERY, OTE, AMX, streptomycin, CIP, norfloxacin, and azithromycin are being used frequently in broiler production in Bangladesh [48]. Bangladesh Government has enacted relevant acts and rules for prudent use of antimicrobials agents through registered veterinarians only for therapeutic purposes in animal production [49,50]. However, the emergence of such AMR due to unscrupulous use of certain antimicrobials agents as growth promoters and feed additives in poultry rearing has now become a significant public health hazard [48,51,52].

In this present study, 78.1% of isolates were found to be multidrug-resistant (MDR) as they demonstrated resistance against 3 to 5 (GEN-ERY-OTE, CHL-ERY-GEN-OTE, AMX-ERY-GEN-OTE, and AMX-CHL-ERY-GEN-OTE) antimicrobial agents. This finding is sparsely supported by a study conducted in Korea as most of the isolates of *C. perfringens* identified from NE outbreaks are found to be R to commonly used antimicrobial agents such as GEN and streptomycin [53]. The phenomenon of MDR development is due to the extensive use of antimicrobial agents as growth promoters and feed additives in poultry feed to prevent Gram-positive bacteria along with *C. perfringens* [54]. In this regard, alternate options such as use of prebiotics and probiotics, herbs, and organic acids, including enzymes, have become more pertinent in poultry production nowadays [44].

The present survey is the first inconclusive research in Bangladesh to evaluate the prevalence, risk factors, and AMR status of *C. perfringens* in broiler flocks. The finding of this research will help in the formulation of proof-based intervention approaches to lessen the burden of *C. perfringens* in poultry, also reduce human introduction of this significant food pathogens.

## Limitations of the study

The main limitation of this study is that the study was conducted only in a single district. However, some important risk factors related to feed composition (like non-starch polysaccharide-enriched feed and use of fish meal) did not consider as a risk factor as the farmers were using high standard commercial poultry feed from the reputed feed companies.

## Conclusion

The study highlights the presence of MDR *C. perfringens* in broiler flocks and its associated burden of treatment failures in poultry and public health hazards. Therefore, evidence-based control options that included improvement of broiler gut health by the use of antibiotic substitutes such as prebiotics, probiotics, and organic acids, including vaccines for NE should be considered. As a part of control measures, biosecurity programs such as proper litter management, dedicated clothing and shoes, and handwashing facilities in each poultry house are needed for lessening the disease occurrence in poultry. Control of coccidia infection in the broiler flock is high demanding for curbing the infection of NE.

## Authors’ Contributions

SMLK: Planned and designed the study. AOT, MA, and AKMZH: Assisted in field survey and laboratory assessment. AOT and SSI: Analyzed the data and drafted the manuscript. SMLK and MTR: Assisted in review and editing of the manuscript. All authors have read and agreed to the published version of the manuscript.
